# Ultrasonic bone curette-assisted unilateral approach for bilateral decompression with MIS-TLIF for severe lumbar spinal stenosis

**DOI:** 10.1186/s12891-024-07453-7

**Published:** 2024-04-23

**Authors:** Yuebing Ren, Ying Nian, Tongxin Sun

**Affiliations:** 1 Department of Spinal Surgery, Dongying People’s Hospital(Dongying Hospital of Shandong Provincial Hospital Group), Dongying, Shandong 257091 China; 2 Department of Oncology, Dongying People’s Hospital(Dongying Hospital of Shandong Provincial Hospital Group), Dongying, Shandong 257091 China; 3 Department of Orthopedics, Dongying People’s Hospital(Dongying Hospital of Shandong Provincial Hospital Group), Dongying, Shandong 257091 China

**Keywords:** Ultrasonic bone curette, Severe lumbar spinal stenosis, Unilateral fenestration, Bilateral decompression, MIS-TLIF

## Abstract

**Purpose:**

We aimed to evaluate the clinical efficacy of bilateral decompression with minimally invasive transforaminal lumbar interbody fusion (MIS-TLIF) assisted by an ultrasonic bone curette (UBC) for treating severe degenerative lumbar spinal stenosis (DLSS) and traditional tool laminectomy decompression MIS-TLIF for treating severe DLSS.

**Methods:**

The clinical data of 128 patients with single-segment severe DLSS who were admitted between January 2017 and December 2021 were retrospectively analyzed. Among them, 67 patients were treated with unilateral fenestration and bilateral decompression MIS-TLIF using an ultrasonic bone curette (UBC group), whereas 61 patients were treated with unilateral fenestration and bilateral decompression MIS-TLIF using traditional tools (traditional group, control). A visual analog scale (VAS) was used to evaluate back and lower limb pain before the operation,immediate postoperative, and one week, 3, 6, 12, and 24 months after the operation. Oswestry disability index (ODI) and Zurich claudication score (ZCQ) were employed to evaluate the improvement in low back and lower limb function. At the last follow-up, the Bridwell bone graft fusion standard was utilized to evaluate bone graft fusion.

**Results:**

The decompression time of laminectomy was significantly shorter in the UBC group than in the traditional group (control group), and the intraoperative blood loss and postoperative drainage volume were significantly less in those in the control group (*P* < 0.05). The VAS, ODI, and ZCQ scores of the two groups after the operation were significantly improved compared to those before the operation (*P* < 0.05). The UBC group had better VAS back scores than the control group immediate postoperative and one week after the operation(*P* < 0.05). The UBC group had better VAS lower limb scores than the control group immediate postoperative (*P* < 0.05).The incidence of perioperative complications, hospitalization time, dural sac cross-sectional area (CSA), and dural sac CSA improvement rate did not differ significantly between the two groups (*P* > 0.05). VAS and ODI scores did not differ significantly between the two groups before,three, six months, one year, and two years after surgery (*P* > 0.05). The ZCQ scores did not differ significantly between the two groups before the operation at one week, six months, one year, and two years after the operation (*P* > 0.05). According to the Bridwell bone graft fusion standard, bone graft fusion did not occur significantly between the two groups (*P* > 0.05) at the last follow-up.

**Conclusions:**

UBC unilateral fenestration bilateral decompression MIS-TLIF in treating severe DLSS can achieve clinical efficacy as traditional tool unilateral fenestration bilateral decompression MIS-TLIF and reduce intraoperative blood loss and postoperative drainage. It can also shorten the operation time, effectively reduce the work intensity of the operator, and reduce the degree of low back pain during short-term follow-ups. Therefore, this is a safe and effective surgical method.

## Introduction

The degenerative lumbar spinal stenosis (DLSS) incidence is increasing annually with the gradual arrival of an aging society, and the number of patients with severe DLSS is also increasing [1]. DLSS is the most common cause of lower back and leg pain in middle-aged and elderly people. The DLSS onset is decreasing due to changes in people’s lives and work styles, and there is a clear trend toward younger age [2]. Severe DLSS is often accompanied by nerve injury symptoms such as lower limb muscle strength and a significant loss of sensation [3]. The effect of conservative treatment is poor or even ineffective, and surgical intervention is often required.

Foley et al. [4] treated lumbar degenerative diseases using minimally invasive transforaminal lumbar interbody fusion (MIS-TLIF) in 2003 and achieved good clinical results. After continuous development, innovation, and improvement of minimally invasive tools, MIS-TLIF surgery technology is maturing, and indications are expanding. Scholars have applied MIS-TILF to treat severe lumbar spinal stenosis and compared it to open transforaminal lumbar interbody fusion or open posterior total laminectomy decompression lumbar interbody fusion. The results exhibit that MIS-TLIF can reduce intraoperative blood loss, shorten hospitalization time, reduce the incidence of adjacent segment degeneration, and achieve excellent interbody fusion and clinical results in the long term [5–7]. However, severe DLSS is frequently accompanied by severe osteoporosis, hyperplasia, and lateral recess stenosis. Traditional surgical methods routinely use osteotomes, lamina bite forceps, and other instruments for laminectomy, resulting in an irregular shape and rough edge of the resected lamina, making it easy to break the dural sac when the lamina is removed, thus increasing the bleeding amount, nerve injury risk, dural sac injury, and complications [7, 8]. The emergence of ultrasonic bone curette technology provides new technical support for reducing the risk of MIS-TLIF surgery.

Ultrasonic osteotome, a new osteotomy tool, has recently been applied to the cervical spine, thoracolumbar, and posterior surgery [9, 10]. The working frequency of the ultrasonic bone curette was 22.5 ~ 40.0 kHz. When the bone tissue reaches its elastic limit under mechanical vibration, it vibrates and cuts [11]. Additionally, mechanical vibration is lower than the elastic limit of soft tissue, thus avoiding mechanical damage to soft tissue [12]. Therefore, the ultrasonic bone curette, a new type of bone tissue-cutting tool, has the advantages of high bone-cutting efficiency, simple operation, especially high tissue selectivity, and unique advantages in spinal cord and nerve root decompression surgeries. However, there are few studies on applying ultrasonic bone curettes in treating severe lumbar spinal stenosis using MIS-TLIF. This study retrospectively analyzed the clinical data of patients with single-segment severe lumbar spinal stenosis who underwent unilateral fenestration and bilateral decompression MIS-TLIF with an ultrasonic bone curette between January 2017 and December 2021. The study compared them with patients who underwent unilateral fenestration and bilateral decompression MIS-TLIF with traditional tools in the same period. This study aimed to explore the therapeutic effects and safety of MIS-TLIF using an ultrasonic bone curette for severe lumbar spinal stenosis.

## Materials and methods

### Study population selection

**Fig. 1:**
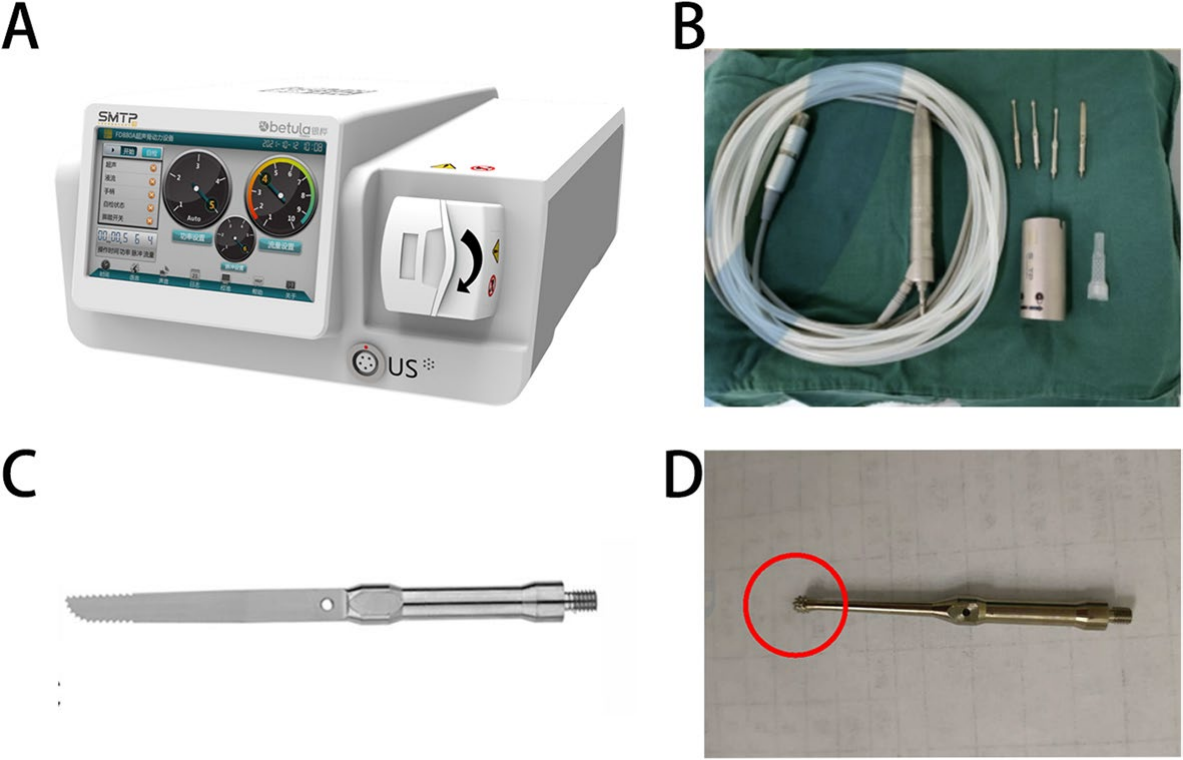
Introduction of Ultrasonic Bone curette Instrument System. **A** Ultrasonic bone dynamic system XD880A. **B** Handle and various types of knife head. **C** Straight blade head. **D** spherical knife head

A retrospective case–control study was conducted on 128 patients diagnosed with severe lumbar spinal stenosis at our hospital between January 2017 and December 2021. All patients underwent MIS-TLIF surgery. Among them, 67 patients underwent MIS-TLIF surgery assisted by an ultrasonic bone curette (XD860A, Jiangsu Shuimu Tianpeng Technology Co. (Fig. 1A, B), Ltd, ultrasonic bone curette group, UBC group), whereas 61 patients underwent MIS-TLIF surgery using traditional instruments (control group). Table 1 summarizes the detailed general data of the two patient groups, which were comparable (*P* > 0.05). All patients in this study met the following inclusion criteria: (1) patients with a significant decrease in muscle strength and sensation of both lower limbs or cauda equina syndrome; (2) patients diagnosed with severe DLSS (Schizas classification according to MRI images, types C and D were diagnosed as severe DLSS) [13]; (3) after regular conservative treatment and improvement of living habits for three months, the symptoms and signs were not relieved or progressively aggravated.The exclusion criteria were as follows: (1) congenital spinal stenosis; (2) severe spinal deformity (scoliosis, kyphosis); (3) combined spinal tuberculosis, trauma, tumor, and other spinal diseases; (4) incomplete follow-up data, follow-up time less than 24 months, and loss to follow-up. This study was approved by the Declaration of Helsinki by the Hospital Ethics Committee. Since this was a retrospective study, informed consent was not required. All data were collected and analyzed anonymously.

**Table 1:** Patients’ demographic data

Variables	UBC (*n* = 67)	Control (*n* = 61)	*p*-value
Age (years)	63.4 ± 10.7	62.9 ± 10.2	0.815
Sex (%)			
Female	49 (73.1%)	43 (70.5%)	0.844
Male	18 (26.9%)	18 (29.5.2%)	
BMI (kg/m^2^)	26.7 ± 3.4	25.7 ± 3.3	0.107
Smoker, n (%)	18 (26.9%)	20 (32.8%)	0.562
Operative level, n (%)			
L3-4	8 (11.9%)	10 (16.4%)	0.769
L4-5	28(41.8%)	24 (39.3%)	
L5-S1	31(46.3%)	27 (44.3%)	
Schizas classification, n (%)			
C	48 (71.6%)	40 (65.6%)	0.567
D	19 (28.4%)	21 (34.4%)	
Duration of disease (days)	36.6 ± 8.5	36.4 ± 8.3	0.889
Comorbidity			
Hypertension	12 (17.9%)	16 (26.2%)	0.289
Cardiopathy	16 (23.9%)	19 (31.1%)	
Lung disease	12 (17.9%)	16 (26.2%)	
Follow-up (months)	26.2 ± 1.6	25.6 ± 2.1	0.097

Schizas classification on MRI

Grade A, CSF is clearly visible inside the dural sac

Grade B, rootlets occupy the entire dural sac but can still be individualized

Grade C, rootlets cannot be individualized with posterior epidural fat and invisible CSF

Grade D, rootlets cannot be individualized without posterior epidural fat

*BMI* body mass index

### Surgical management

**Fig. 2:**
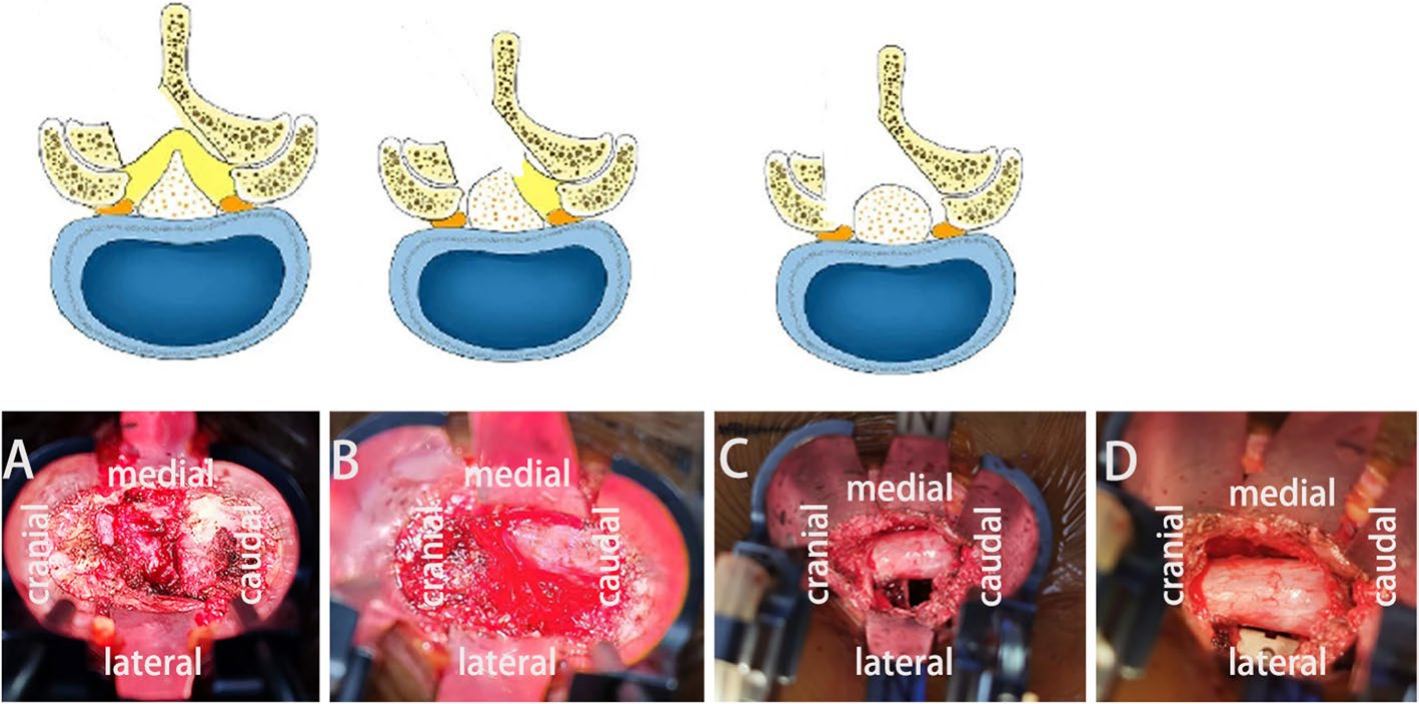
The operation process of ultrasonic bone curette MIS-TLIF technique. **A** The ultrasonic bone curette was used to remove the vertebral plate and part of the inferior articular process. **B** The ultrasonic bone curette was used to remove the whole inferior articular process, the part of the superior articular process,spinous process root and ipsilateral ligamentum flavum,fully exposed the ipsilateral outlet root and the running root. **C** The ultrasonic bone curette was utilized to remove the contralateral lamina,the lamina rongeur was employed to remove the contralateral ligamentum flavum,while the ultrasonic bone curette(spherical knife head) was used to expand the contralateral nerve root canal and lateral recess, and the contralateral nerve root was fully released. **D** The bilateral nerve roots were completely released and bone graft was performed. The interbody fusion cage was inserted

The same senior surgeon performed all surgeries. After administering general anesthesia, the patient was placed in the prone position, a C-arm fluoroscopy was used to locate the surgical segment (and mark the pedicle surface projection position), and the surgical incision was marked with a marker pen. The towel was conventionally disinfected, the skin layer by layer, deep and superficial fascia was cut, blunt separation along the muscle gap established a working channel, and the upper and lower articular processes and part of the lamina were fully exposed. The specific decompression steps in the two groups were as follows. Both groups were treated with a minimally invasive pedicle screw system for bilateral percutaneous screw fixation.

ultrasonic bone curette group (UBC, group): When using the ultrasonic bone curette(Straight blade head, Fig. 1C) to remove the lamina, the ultrasonic bone curette should be perpendicular to the bone surface as far as possible, so as to avoid damaging the head of the ultrasonic bone curette and prolong its service life.When performing contralateral decompression, a special ultrasonic bone curette head needs to be used(such as spherical knife head Fig. 1D).The working channel was established on the side with severe lower limb symptoms, and an ultrasonic bone curette was utilized to remove the inferior articular process, and the part of the superior articular process fully exposed the ipsilateral outlet root and the running root (Fig. 2A). When performing contralateral decompression, a special ultrasonic bone curette head needs to be used.An ultrasonic bone curette was deployed to remove the side of the lamina from the root of the spinous process, while a lamina rongeur was used to remove the ipsilateral ligamentum flavum. The nerve stripper was employed to moderately press the dura mater, whereas the contralateral ligamentum flavum was removed. An ultrasonic bone curette was used to expand the contralateral nerve root canal and lateral recess, and the contralateral nerve root was fully released. The intervertebral disc was treated (fully scraped the cartilage endplate), autologous bone particles were implanted in the bone graft funnel (supplemented with allogeneic bone if necessary), and the intervertebral fusion device was placed (Fig. 2B-D).

Traditional group (control group): The working channel was established on the side with severe lower-limb symptoms. The lower articular process and part of the upper articular process were removed using the traditional osteotome, and the ipsilateral outlet root and running root were fully exposed. The intervertebral disc was treated (the cartilage endplate was fully scraped). Autologous bone particles were implanted in the bone graft funnel (allogeneic bone was supplemented if necessary), and the intervertebral fusion cage was placed. A traditional osteotome or vertebral plate bone rongeur was employed to remove one side of the lamina from the root of the spinous process. A vertebral plate bone rongeur was utilized to remove the ipsilateral ligamentum flavum, while the nerve stripper was used to press the dura mater moderately to remove the contralateral ligamentum flavum. A vertebral plate bone rongeur was used to expand the contralateral nerve root canal and lateral recess to fully release the contralateral nerve root.

### Postoperative management

The postoperative drainage volume and lower limb activity were observed to prevent hematoma formation and nerve compression during the incision. The drainage tube was removed when the drainage volume was less than 50 mL within 24 h. Patients with cerebrospinal fluid leakage were given bedside elevation, and the drainage tube was intermittently clipped after the drainage fluid color was apparent. The drainage tube was removed between the third and fifth postoperative days, sutured, and pressurized. After extubation, X-rays, CT, and MRI were performed to evaluate decompression and internal fixation. A venous ultrasound of both lower extremities was used to exclude venous thrombosis of the lower extremities, and a thoracolumbar brace was worn to get out of bed.

### Evaluation criteria

In this study, three independent authors collected clinical data, including clinical and imaging evaluation results before surgery, one week, 1, 3, 6, 12, and 24 months after surgery, and at the last follow-up. All patients were followed up for more than two years.

**Fig. 3:**
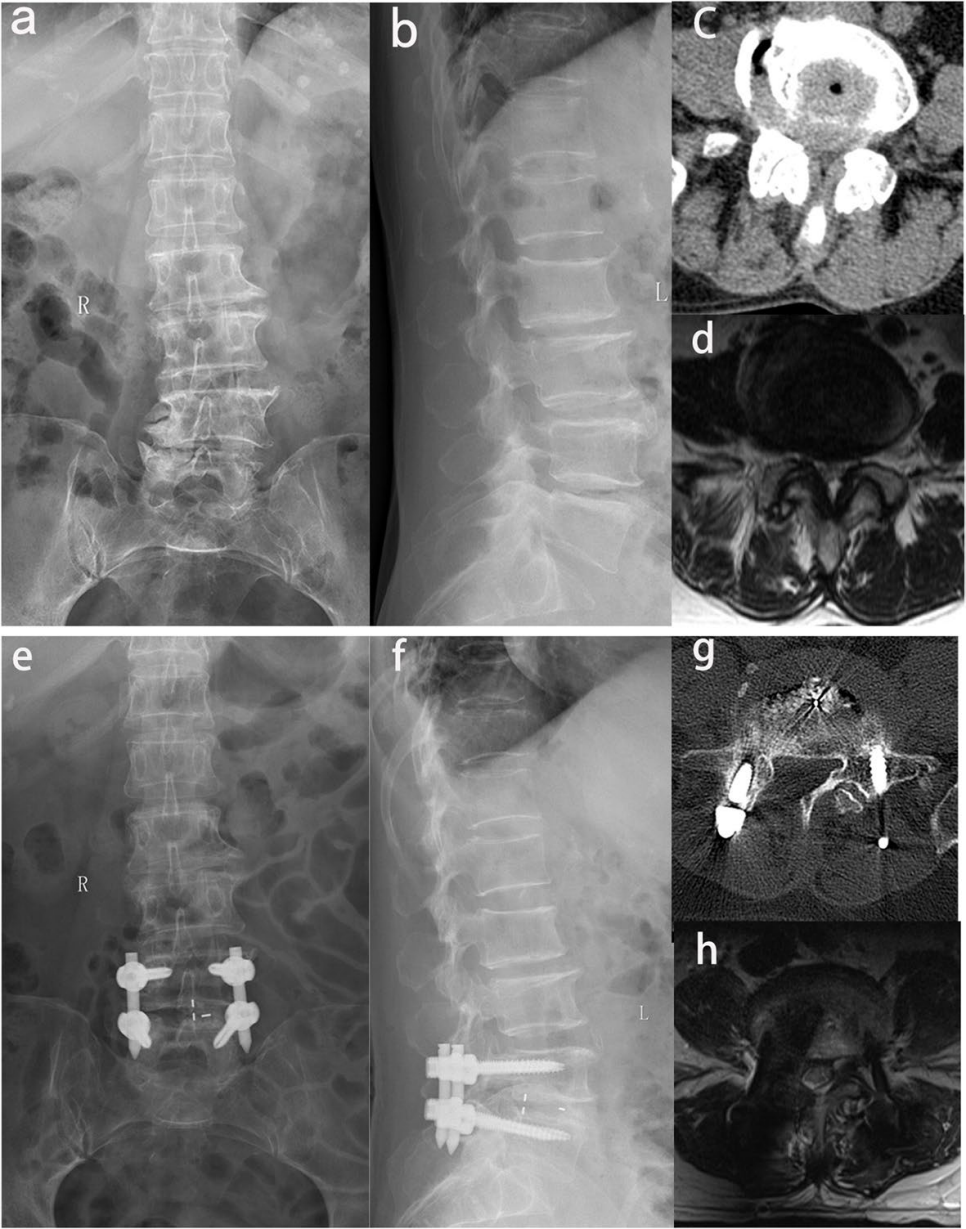
In the group of ultrasonic bone curette, a 74-year-old woman suffered from low back pain accompanied by intermittent claudication for more than three years. Preoperative X-ray (**a**, **b**), MRI (**d**), and CT (**c**) examinations showed severe DLSS (Schizas Grade D) at the L4/L5 level. The patient received Mis-TLIF assisted by an ultrasonic bone curette, and symptoms were significantly relieved after the surgery. Postoperative X-ray (**e**, **f**) and postoperative CT/MRI indicated that complete decompression was achieved at L4/L5 (**g**, **h**)

Clinical evaluation and imaging evaluation index: (1) Visual analog scale (VAS): The pain VAS scores of the patients were measured by nurses and two doctors in the same group before the operation, one week after the operation, and at each follow-up time point. The average value was calculated and recorded. (2) The Oswestry disability index (ODI) and the Zurich Claudication Questionnaire (ZCQ) were calculated by three doctors in the same group before the operation, one week after the operation, and at each follow-up time point. The average value was calculated and recorded. (3) The hospitalization time, operation time, intraoperative blood loss, and perioperative complications were recorded, including incision complications such as infection, incision nonunion, hematoma formation, internal fixation complications such as pedicle screw misplacement, endplate fracture, cage subsidence and displacement, pedicle screw loosening and fracture, dural and nerve root complications such as an intraoperative tear of the dural sac, injury of a nerve root or cauda equina nerve, and contralateral nerve symptoms. (4) Fusion rate: at the last follow-up, the lumbar spine was collected, and the lumbar interbody fusion was graded using the Bridwell method [14]. I and II were fusions. If DR examination cannot be performed, a lumbar CT examination can be further improved.Typical cases are shown in Fig. 3.

### Statistical analysis

All data are expressed as mean ± standard deviation unless otherwise specified. A board-certified spine surgeon blinded to the procedure evaluated all radiographic results. Interobserver reliability was assessed using intraclass correlations with data measured by one of the co-authors and classified as poor (0–0.39), moderate (0.40–0.74),or excellent (0.75–1.00). For continuous variables, within-group and between-group differences were detected using Student’s and paired t-tests, respectively. Chi-square analysis was performed to compare categorical variables.Statistical significance was set at *P* < 0.05. All statistical analyses were performed using the SPSS software (version 23.0; SPSS Inc., Chicago, IL, USA).

## Results

Table 1 summarizes the demographic and baseline characteristics of the two groups. Baseline demographic data analysis revealed no significant differences between the two groups (*P* > 0.05). The two groups were primarily Schizas grade C (severe stenosis, UBC 71.6%, control 65.6%), and the remaining patients had severe stenosis (Schizas grade D). The course, combined disease, and follow-up time did not differ significantly between the two groups.

The UBC group had a shorter operation time (146.9 ± 13.9 vs.152.1 ± 13.7 min) and less estimated blood loss (116.9 ± 16.5 vs. 41.5 ± 22.2 mL) than the control group, and the difference between the two groups was statistically significant (*P* < 0.05, Table 2). The length of hospital stay did not differ significantly between the two groups (*P* > 0.05). The perioperative complications of dural sac tear (*n* = 2) in the UBC group and dural sac tear (*n* = 3) and incision infection (*n* = 1, *n* = 2) in the control group did not differ significantly (*P* > 0.05). These complications subsided within one month of the operation. CSA did not differ significantly between the two groups after operation (*P* > 0.05), confirming that the decompression effect of the two groups after operation (UBC preoperative 0.82 ± 0.09 vs. postoperative 1.53 ± 0.05 cm^2^, control preoperative 0.81 ± 0.08 vs. postoperative 1.54 ± 0.04 cm^2^, *P* < 0.05, Table 2) was equivalent.

**Table 2:** Perioperative characteristics by type of procedure

Variables	UBC (*n* = 67)	Control (*n* = 61)	*p*-value
Operative time, min	146.9 ± 13.9	152.1 ± 13.7	0.035^*^
EBL, mL	116.9 ± 16.5	123.8 ± 15.8	0.017^*^
Length of hospital stay (days)	5.6 ± 1.7	5.8 ± 1.5	0.551
Perioperative complications, n (%)	3	5	0.605
Dural sac tearing	2 (3%)	3 (4.9%)	
Incision infection	1	2 (3.4%)	
Dural sac CSA, cm^2^			
Preop	0.82 ± 0.09	0.81 ± 0.08	0.700
1yr postop	1.53 ± 0.05	1.54 ± 0.04	0.447
Improvement percentage of dural sac CSA (%)	87.7 ± 20.3	89.9 ± 20.8	0.546

*EBL* estimated blood loss, *CSA* cross-sectional area

^*^indicates *p* < 0.05

The VAS, ODI, and ZCQ scores of the two groups were significantly improved at each follow-up time point than before the operation (*P* < 0.05). Six hours after the operation, the VAS back and leg (2.94 ± 0.74,3.36 ± 0.77) scores were significantly better in the UBC group than in the control group (VAS back: 3.16 ± 0.90, VAS leg:3.67 ± 0.81, *P* < 0.05, Table 3). One week after the operation, the VAS back (2.82 ± 0.76) scores were significantly better in the UBC group than in the control group (VAS: 3.16 ± 0.90, *P* < 0.05, Table 3). The VAS and ODI scores did not differ significantly between the two groups at three and six months, one year, and two years after surgery (*P* > 0.05). The ZCQ scores did not differ significantly between the two groups at any follow-up time point (*P* > 0.05).

**Table 3:** Comparison for postoperative VAS, ODI, and ZCQ score

Scoring System	UBC (*n* = 67)	Control (*n* = 61)	*p*-value
VAS back			
Preop (mean score)	6.95 ± 1.51	7.00 ± 1.53	0.868
Postop (6 h)	2.94 ± 0.74	3.36 ± 0.77	0.002^**^
Postop (1 wk)	2.82 ± 0.76	3.16 ± 0.90	0.021^*^
Follow-up at 6 mos	2.36 ± 0.83	2.34 ± 0.73	0.920
Follow-up at 1 yrs	1.94 ± 0.69	2.11 ± 0.61	0.134
Follow-up at 2 yrs	1.82 ± 0.60	1.93 ± 0.54	0.266
*p*-value (pre vs. post)	0.000^**^	0.000^**^	
VAS leg			
Preop (mean score)	7.13 ± 1.47	7.25 ± 1.41	0.662
Postop (6 h)	3.34 ± 0.75	3.67 ± 0.81	0.019^*^
Postop (1 wk)	3.18 ± 0.72	3.39 ± 0.86	0.127
Follow-up at 6 mos	2.21 ± 0.83	2.18 ± 0.72	0.835
Follow-up at 1 yrs	1.69 ± 0.63	1.77 ± 0.53	0.420
Follow-up at 2 yrs	1.64 ± 0.60	1.72 ± 0.52	0.413
*p* value (pre vs post)	0.000^**^	0.000^**^	
ODI			
Preop (mean score)	62.13 ± 3.51	61.13 ± 2.82	0.079
Follow-up at 3 mos	31.62 ± 5.44	33.15 ± 4.80	0.097
Follow-up at 6 mos	16.58 ± 2.40	16.77 ± 2.41	0.659
Follow-up at 1 yrs	12.81 ± 1.36	12.97 ± 1.48	0.523
Follow-up at 2 yrs	12.10 ± 0.92	12.23 ± 0.88	0.236
*p*-value (pre vs. post)	0.000^**^	0.000^**^	
ZCQ			
Preop (mean score)	66.85 ± 2.57	66.03 ± 2.31	0.062
Postop (1 wk)	38.67 ± 3.67	39.85 ± 3.90	0.080
Follow-up at 6 mos	22.18 ± 3.10	21.59 ± 2.72	0.258
Follow-up at 1 yrs	19.72 ± 2.39	20.20 ± 1.97	0.219
Follow-up at 2 yrs	18.71 ± 1.74	19.10 ± 1.77	0.220
*p*-value (pre vs. post)	0.000^**^	0.000^**^	

*VAS* Visual Analogue Scale, *ODI* Oswestry Disability Index, *ZCQ* Zurich Claudication Questionnaire

^*^indicates *p* < 0.05

^**^indicates *p* < 0.01

## Discussion

The ultrasonic bone curette converts the electrical signal into a mechanical vibration via a piezoelectric converter, causing the knife head to vibrate at a high frequency and low amplitude. Due to the difference in tissue density and elasticity, most of the energy generated by the ultrasonic bone curette is absorbed by hard bone tissue. They play a role in bone cutting via mechanical fragmentation and cavitation. Soft tissues, such as the nerve roots, dura mater, and spinal cord, are in elastic contact with the ultrasonic scalpel at the same frequency and amplitude, reducing the risk of direct damage to soft tissues [9, 15]. Studies have demonstrated that ultrasonic bone knives can effectively avoid mechanical and thermal damage to nerve roots compared to high-speed grinding drills or traditional tools, such as vertebral plate bone rongeurs, bone knives, and pointed mouth bone rongeurs [16, 17]. Morimoto et al. [11] discovered that the ultrasonic osteotome for lumbar lamina fenestration and intervertebral foramen decompression in treating lumbar spinal stenosis had less surgical trauma and higher surgical safety than traditional osteotomes and lamina rongeurs. This study discovered that, in clinical practice, an ultrasonic bone curette outperforms a high-speed grinding drill and a gun-shaped bone rongeur for spinal canal decompression. Severe DLSS mostly has ossified hard ligamentum flavum or intervertebral discs and severe bone hyperplasia. The pressure-causing substance is closely adhered to the dural sac and nerve root with no buffer space [1, 2]. If a high-speed grinding drill and a gun-shaped bone rongeur are used for decompression, a brain cotton sheet and nerve stripper must be used to depress and protect the dura mater. The above operation of traditional tools in narrow spaces increases the difficulty and time of the operation and increases the risk of nerve and dura mater injury. When a high-speed grinding drill is used, there is a winding of soft tissue, and the narrow space is unclear, which can easily cause the risk of dural and nerve root injury. ultrasonic bone curette decompression requires only forcing the rake head to the back and grinding the bone structure, thereby reducing the risk of dural compression and nerve root injury. This study revealed that the postoperative dural sac CSA of the two groups was significantly higher than before surgery, with no significant difference in postoperative CSA between the two groups. Nerve root injury has no complications in the two groups. There were two cases of dural injury and three cases of cerebrospinal fluid leakage in the UBC and control groups, respectively. The incidence between the two groups did not differ significantly. Our operational experience is that the safety of ultrasonic bone curette decompression is higher than that of traditional tools, but our study is a retrospective study, with fewer cases included. For the comparison of the incidence of complications of dural tear and nerve root injury between the two groups, a multi-center prospective study is still needed for further study.Therefore, we believe that an ultrasonic bone curette is a safe and feasible tool for laminectomy, lateral recess decompression, and decompression surgery.

Previous reports have achieved good clinical results using a gun-shaped bone rongeur, high-speed grinding drill, or endoscopic unilateral fenestration to sneak out the hypertrophic ligamentum flavum, expand the lateral recess and nerve root canal, and expose and release the contralateral nerve root [18, 19]. In this study, patients in the UBC group were treated with decompression of the contralateral spinal canal and nerve root canal using direct vision with an ultrasonic bone curette. Postoperative low back pain, intermittent claudication, and low back dysfunction were significantly relieved compared to those before the operation. The VAS, ODI, and ZCQ scores did not differ significantly between the two groups during the mid-term follow-up (*P* > 0.05). Our study presented no significant difference in intervertebral fusion rate between the two groups at the last follow-up (*P* > 0.05). Therefore, we believe that MIS-TLIF with unilateral fenestration and bilateral decompression using an ultrasonic bone curette can achieve mid-term clinical efficacy similar to that of the traditional tool laminectomy decompression.

Studies have demonstrated that the high-frequency vibration of the contact surface between the blade and bone tissue can produce thermal and cavitation effects during the removal of the lamina by the ultrasonic osteotome, thereby significantly reducing the bleeding of the bone tissue section [20]. The instantaneous high temperature generated at the interface between the ultrasonic bone curette and the bone promotes the contraction of local microvessels and increases thrombin activity, playing an important role in local hemostasis [20, 21]. The cavitation effect of the ultrasonic bone curette strengthens the local coagulation function by emulsifying and breaking the surrounding soft tissue and promoting coagulation and degeneration of hemoglobin [15, 20, 21]. Bone wax is frequently used to stop bleeding from the bone surface when traditional bone knives and gun-shaped bite forceps are used to remove the lamina. This method of hemostasis is rough and often incomplete, which is an important factor in increasing the amount of intraoperative bleeding. Studies have revealed that using an ultrasonic bone curette for cervical laminectomy, thoracic laminectomy, and osteotomy in the scoliosis correction steps to treat the corresponding diseases results in less intraoperative blood loss than traditional laminectomy tools [15, 20–22]. In this study, the ultrasonic bone curette group had significantly less intraoperative blood loss and postoperative drainage volume than the traditional group, consistent with previous reports. Therefore, we believe that an ultrasonic bone curette is important to reduce bleeding during lumbar laminectomy and lateral recess decompression.

DLSS is more common in middle-aged and elderly patients. It is easy to cause clinical symptoms, such as lower back pain, intermittent claudication, and radiation pain in both lower limbs, seriously affecting the patient’s quality of life. MIS-TLIF is the most widely used and mature minimally invasive decompression and fusion technique. Unilateral fenestration and bilateral decompression mis-TLIF have been used in the surgical treatment of DLSS and have achieved good clinical results [22, 23]. However, severe DLSS mostly has ossified hard ligamentum flavum or intervertebral discs and severe bone hyperplasia. The dural sac and nerve root lack movement space, and the pressure substance is closely adhered to the dural sac and nerve root, often without any buffer space. Conventional decompression tools can easily damage the dural sac and the nerve roots. A bone knife, lamina bite forceps for narrow segment laminectomy, nerve root canal enlargement, unilateral approach bilateral nerve root decompression, or bilateral establishment of channels for bilateral decompression are used in conventional MIS-TLIF surgery for severe lumbar spinal stenosis [22–24]. This type of surgery involves large trauma, more bleeding, and longer operation time, which can easily increase the incidence of perioperative complications [23, 24]. The operation time and bone-cutting efficiency of the ultrasonic osteotome were significantly better than those of traditional tools. In this study, the operation time of the ultrasonic osteotome group was significantly shorter than that of the traditional group, which is consistent with previous reports [20, 21]. To solve the above problems, we adopted the ultrasonic bone curette-assisted unilateral approach of bilateral decompression MIS-TLIF surgery to treat lumbar spinal stenosis, reduce intraoperative bleeding, shorten the operation time, and obtain good clinical efficacy. However,the difference of operation time and estimated blood transfusion volume between the two groups is small which may be related to the small number of included cases,multicenter prospective study is needed to further verify.In this study, the VAS back and leg scores were better in the ultrasonic bone curette group than in the traditional group six hours after the operation, possibly due to the stimulation and concussion of peripheral nerves by conventional tools (bone knife and vertebral plate bone biting forceps) in the traditional group. The VAS, ODI, and ZCQ scores did not differ significantly between the two groups during the mid-term follow-up (*P* > 0.05). Therefore, we believe that MIS-TLIF assisted by an ultrasonic bone curette has less trauma, faster recovery, and shorter operation time than the traditional tool group while achieving the same mid-term clinical efficacy. The long-term clinical efficacy requires further follow-up. However, compared with traditional tools, ultrasonic bone curette was expensive. For beginners, it was easy to damage ultrasonic bone curette because of the inappropriate decompression method and this increases the cost of surgery and the economic burden of patients.This was a retrospective clinical study. The evidence level was low, and the total number of cases was small. Prospective clinical studies with larger sample sizes are required.

## Conclusion

Ultrasonic osteotome unilateral fenestration bilateral decompression MIS-TLIF for treating severe DLSS can achieve mid-term clinical efficacy similar to that of traditional tool laminectomy decompression MIS-TLIF. It can reduce operative time, intraoperative blood loss, and postoperative drainage. Short-term follow-up can reduce the incidence of low back pain. Therefore, this is a safe and effective surgical method. However, this was a retrospective clinical study. The evidence level was low, the sample size was small, and a prospective clinical study of larger cases is needed to prove further.

## Data Availability

The datasets used and/or analysed during the current study are available from the corresponding author on reasonable request.

## Abbreviations

DLSS: Degenerative Lumbar Spinal Stenosis
MIS-TLIF: Minimally Invasive Transforaminal Lumbar Interbody Fusion
UBC: Ultrasonic bone curette
VAS: Visual Analogue Scale
ODI: Oswestry disability index
ZCQ: Zurich claudication score
MRI: Magnetic resonance imaging
